# LASP1 promotes proliferation, metastasis, invasion in head and neck squamous cell carcinoma and through direct interaction with HSPA1A

**DOI:** 10.1111/jcmm.14854

**Published:** 2019-12-02

**Authors:** Qi Chen, Kun Wu, Xing Qin, Youcheng Yu, Xu Wang, Kuijie Wei

**Affiliations:** ^1^ Department of Stomatology Qingpu Branch of Zhongshan Hospital Fudan University Shanghai China; ^2^ Department of Oral and Maxillofacial‐Head & Neck Oncology Ninth People's Hospital Shanghai Jiao Tong University School of Medicine Shanghai China; ^3^ Department of Stomatology of Zhongshan Hospital Fudan University Shanghai China

**Keywords:** HNSCC, HSPA1A, LASP1, metastasis, proliferation

## Abstract

LIM and SH3 protein 1 (LASP1) is a specific focal adhesion protein that promotes metastasis in a variety of tumours. However, its role in head and neck squamous cell carcinoma (HNSCC) has not been fully validated. The purpose of this study was to analyse the interaction of LASP1 and its binding partner in HNSCC. The expression of LASP1 and HSPA1A in HNSCC was analysed by real‐time PCR and Western blot. The effects of LASP1 on the biology behaviour of HNSCC cell lines were observed in vivo and in vitro. Co‐immunoprecipitation analysis was performed to confirm the interaction between LASP1 and HSPA1A. LASP1 was highly expressed in HNSCC and associated with poor prognosis for patients. LASP1 also promoted cell proliferation, colony formation, invasion and cell cycle G2/M phase transition. Heat shock protein family A member 1A (HSPA1A) is identified as a chaperone protein of LASP1 and co‐localized in the cytoplasm. HSPA1A positively regulates the interaction of LASP1 with P‐AKT and enhances the malignant behaviour of HNSCC cells. LASP1 and HSPA1A are both up‐regulated in HNSCC, and directly binds to each other. Double inhibition of LASP1 and HSPA1A expression may be an effective method for the treatment of HNSCC.

## 1 INTRODUCTION

LIM and SH3 protein 1 (LASP1) is a gene originally screened from metastatic lymph nodes in breast cancer and is identified as a nuclear sputum shuttle protein.^1^ LASP1 can mediate tumour growth, metastasis and invasion in many malignant tumours.^2^, ^3^, ^4^ Its expression level is closely related to tumour size and malignancy, especially lymph node metastasis of tumours.^5^, ^6^ LASP1 functions as a chaperone protein of chemokines and backbone proteins,^7^, ^8^ promoting the signal cascade in PI3K/AKT, MAPK and other signalling pathways via elevated phosphorylation, especially in gallbladder cancer; LASP1 promotes biological behaviour by down‐regulating S100P via the PI3K/AKT pathway.^9^, ^10^

Heat shock protein family A member 1A (HSPA1A) is a member of the heat shock protein 70 family. In conjunction with other heat shock proteins, HSPA1A mediates the folding of newly translated protein and makes existing proteins stable against aggregation in the cytosol and in organelles.^11^ HSP70 stabilizes the production of a large number of oncogenic proteins and ultimately supports tumour growth and survival in the pathogenesis of cancer.^12^, ^13^, ^14^ HSPA1A is associated with increased cancer cell proliferation, increased tumour grade and reduced overall survival.^15^ Extracellular HSPA1A promotes tumour cell proliferation and apoptosis resistance in liver.^16^ HSPA1A has high secretion in ovarian cancer, and the amount of secretion depends on different tumour types of ovarian cancer.^17^ However, the relationship between LASP1 and HSPA1A has not been well examined.

In this present study, we found that HSPA1A directly binds with LASP1, co‐located in the cell cytoplasm and plays a synergistic role in promoting tumours in HNSCC by elevating the interaction between LASP1 and P‐AKT. Our results suggested that LASP1 promoted HNSCC proliferation, metastasis and invasion through direct interaction with HSPA1A.

## 2 METHODS AND MATERIALS

### 2.1 Patients

The clinical samples used in this study are 40 pairs of HNSCC tissues and adjacent normal tissues. All the samples were collected from the Department of Oral and Maxillofacial‐Head and Neck Oncology, Ninth People's Hospital, Shanghai Jiao Tong University School of Medicine (Shanghai, China).

### 2.2 Cell culture

The HNSCC cell lines HN4, HN6 and HN30 were obtained from the University of Maryland Dental School, USA, and CAL‐27 was provided by the American Type Culture Collection, USA. The periodontal ligament cells (PDLC) which are epithelial cells from head and neck in this study were primarily cultured in DMEM supplemented with 10% heat‐inactivated FBS, penicillin (100 units/mL) and streptomycin (100 μg/mL).^18^ The SCC‐25 cell line was purchased from the American Type Culture Collection, USA and was cultured in DMEM/F12 medium containing 10% FBS. All cell lines were cultured in a humidified, 5% CO_2_ incubator at 37°C.

### 2.3 RNA extraction and real‐time PCR

Total RNA was isolated from head and neck tumour tissues and cell lines according to the manufacturer's instructions (9108/9109) and measured at optical density measurements at 260 nm and 280 nm for total RNA concentration and purity; the RNA was then converted to cDNA using a reverse transcriptase kit (DRR420A). Experiments were performed using the real‐time PCR instrument (ABI PRISM 7500). The primers were synthesized by Shanghai Sheng Gong Bioengineering Co., Ltd., and the sequence was as follows: LASP1: F: 5′‐CTG TCT CTG CCT TAT AGC AAC AC‐3′, R: 5′‐CAT CTC GAA CCT GGC TGT TTG‐3′. HSPA1A: F: 5′‐CCA GAA CAA GCG AGC CGT GAG‐3′, R: 5′‐TCG GAA CAG GTC GGA GCA CAG3′. GAPDH: F:5′‐CAT CTC TGC CCC CTC TGC TGA‐3′, R: 5′‐GGA TGA CCT TGC CCA CAG CCT‐3′. Three replicate wells were set in each group, GAPDH was used as an internal reference, and the relative expression level of the gene was calculated using 2^‐ΔΔCt^.

### 2.4 Western blot analysis

The total protein of HNSCC cells and PDLC cells was extracted with SDS lysis buffer (Beyotime) and determined by BCA kit (Thermo). The amount of protein in each swimming lane was 30 µg in the premade gel (M01010C, Shanghai Jinsirui Biotechnology Co, Ltd, China Catalog number) and was electrophoresed at a constant pressure of 140 V for 1 hours, followed by a transmembrane with a constant 400‐mA flow. The PVDF membrane (Merck Millipore) was incubated with primary antibody at 4°C overnight. The antibodies are listed as follows: a rabbit anti‐human LASP1(8636), polyclonal primary antibody; rabbit anti‐human P‐AKT (4060), polyclonal primary antibody; rabbit anti‐human AKT (2920), polyclonal primary antibody; rabbit anti‐human β‐tublin (2148), polyclonal primary antibody (Cell Signaling Technology); and rabbit anti‐human HSPA1A, polyclonal primary antibody (10995‐1‐AP, Proteintech). HRP‐labelled rabbit antibody (7074, Cell Signaling Technology) was used for incubation for 1h, and an ECL (WBKLS0100, Millipore) developing solution was added and developed on a chemiluminometer (Amersham Imager 600).

### 2.5 Lentivirus construction and infection

LASP1 interference control lentivirus and three interfering lentiviruses, overexpression control and overexpression lentivirus were constructed by Shanghai Jiman Biotechnology Co, Ltd. The target strand sequences of human shRNAs for LASP1 and HSPA1A were as follows: LASP1, shRNA1: GAA CTA CAA GGG CTA CGA GAA; shRNA2: GCA TAA AGC ATG CTT CCA TTG; shRNA3: GCG CTA CAA GGA GGA GTT TGA; NC shRNA: TTC TCC GAA CGT GTC ACG T. HSPA1A: shRNA1: GGA TCC AGT GTT CCG TTT CCA; shRNA2: GTC AGT TCT CAA TTT CCT GTG; shRNA3: GCC ATC TTA CGA CTA TTT CTT; NC shRNA: TTC TCC GAA CGT GTC ACG T. The cDNA sequence of LASP1 is in accordance with the Genebank (PUBMED ID: NM 001271608.1) and the transduct into PGMLV‐6751. The empty lentiviruses were selected as the negative control for OE‐DNA.

The average 1 × 10^5^ cells CAL27 and HN6 cells per well were seeded in the six‐well plate, cultured until the cell density was about 40%. After 24 hours of infection, the complete medium was replaced. The infection efficiency was observed after 48 hours, and the cells were screened with puromycin‐containing medium after 72 hours. Two weeks later, CAL27, HN6 interference and overexpression stable cell lines were constructed. The HSPA1A interfering cell line was constructed in the same manner in the CAL27 (sh‐LASP1 and OE‐LASP1) and HN6 (sh‐LASP1 and OE‐LASP1) stable cell lines using the blasticidin.

### 2.6 Cell proliferation assay

The cells in logarithmic growth phase were inoculated into the 96‐well plates at 1000 cells/well. The CCK‐8 reagent (DOJINDO, Japan) was added at 10 µL per well and placed in the incubator for 2 hours, and then, it was detected by a microplate reader (Molecular Devices, SpectraMax i3) at a wavelength of 450 nm. Absorbance (OD) values were recorded to indicate the cell viability.

### 2.7 Plate colony formation assay

The cells in logarithmic growth phase were inoculated into six‐well plates at 500 cells/well, and the culture medium was changed every two days. When the cells showed obvious clonal proliferation, the culture was ceased. The culture plates were washed three times with PBS, fixed with paraformaldehyde for 30 minutes and washed with PBS for another three times. The cells were stained with 0.1% crystal violet staining for 1 hours, followed by wash with PBS three times. The images were recorded with a scanner (CanoScan 5600F), which quantified the number of cell clones with over 50 cells.

### 2.8 In vitro cell migration and invasion assay

The cells in the logarithmic growth phase were cultured in a double‐free (serum‐free and antibiotic‐free) DMEM medium and then inoculated into the transwell chamber (pores 0.8 µm, Merck Millipore) at the density of 5 × 10^5^/well. The cell invasion assay was implemented with Matrigel (BD Biosciences) in chamber. After culturing for 24 hours, the cells were fixed and stained with 0.1% crystal violet for 1 hour, followed by washing with PBS 3 times. Images of three fields were captured, and the cells were counted.

### 2.9 Cell cycle analysis

The 2 × 10^5^ cells in logarithmic growth phase were seeded into each well of the six‐well plates for 24 hours. The cells were collected and fixed in 70% ethanol for 24 hours at 4°C overnight, followed by washing with PBS 3 times. Next, cell cycle detection reagent (556547) was used to stain for 0.5 hours on ice, and the cells were analysed by flow cytometry (BD FACS Calibur).

### 2.10 In vivo tumour invasion assay

All animal experiments were approved by the Animal Research Committee of the Experimental Animal Research Center of the Ninth People's Hospital of Shanghai and in accordance with the guidelines for international animal research. Tumour cells CAL27 (Scramble or sh‐LASP1) (1 × 10^6^) were injected into nude mice through the tail vein to evaluate the tumorigenic ability. Five mice were used in each group. After 42 days, the mice were sacrificed by cervical dislocation. The number of metastatic lung nodules in each mouse was counted under the microscope (AXIO, Scope.A1). H‐E staining and immunohistochemistry (1:50, Cell Signaling Technology) were used to analyse tumours in the lung, followed by a statistical analysis of the differences between the CAL27 scramble control and interference groups.

### 2.11 Immunofluorescence

When the CAL27 and HN6 cells were cultured on coverslips with 40% density, the cells were fixed with 4% paraformaldehyde for 1 hour and then incubated with 0.1% Triton X‐100 for 5 minutes. After the cells were blocked in goat serum for 0.5 hour, the cells were incubated with LASP1 antibody (1:100, Cell Signaling Technology) and HSPA1A antibody (1:100, Proteintech) at 4°C overnight. The following day, the slips were incubated with the corresponding secondary antibody. The cells were contained with 4′,6‐diamidino‐2‐phenylindole (DAPI; 1:300, Invitrogen) to detect nuclei. The cells were observed and imaged using a TCS SP2 laser‐scanning confocal microscope (Leica Microsystems).

### 2.12 Co‐immunoprecipitation (Co‐IP) analysis

The cells were lysed with IP buffer (20118ES60, Shanghai Yeasen Biotechnology Co, Ltd). After the protein concentration was determined to be 1 mg/mL, the cell lysates were incubated with rabbit anti‐human LASP1(8636, CST) or HSPA1A (10995‐1‐AP, Proteintech) antibody, as well as the protein A/protein G‐coated agarose beads (Merck) overnight at 4°C; the cell lysates were then washed with PBS 3 times and centrifuged each time at 1000 × *g* for 30 seconds, and the proteins were then separated from the beads using immunoblotting loading buffer for 10 minutes at 95°C. The supernatants were collected for subsequent immunoblotting analysis with the indicated antibodies.

In the LC/MS/MS assay, CAL27(OE‐LASP1) cells were lysed with IP buffer, and anti‐FLAG beads (M8823, Sigma) were added into the cell lysates at 4°C overnight; as mentioned above, we used Protein Stains Kit (C500021‐0010, Shanghai Sangon Biotech Co, Ltd) and noticed an obvious band, and this band was analysed by (Shanghai Luming Biotech Co, Ltd).

### 2.13 Statistical analysis

The data were analysed using SPSS19.0. The interrelationship between LASP1 expression and histology or clinical factors was analysed using Fisher's exact test and χ^2^ tests of independence. The Kaplan‐Meier test was used for univariate survival analysis. The Cox regression test was used for multivariate analyses. Student's *t* test and one‐way ANOVA were used to compare the means of 2 groups or more. All of the experiments were independently performed 3 times. *P* < .05 was considered as significant.

## 3 RESULTS

### 3.1 LASP1 is up‐regulated in HNSCC and is associated with the poor prognosis of patients

**Figure 1:**
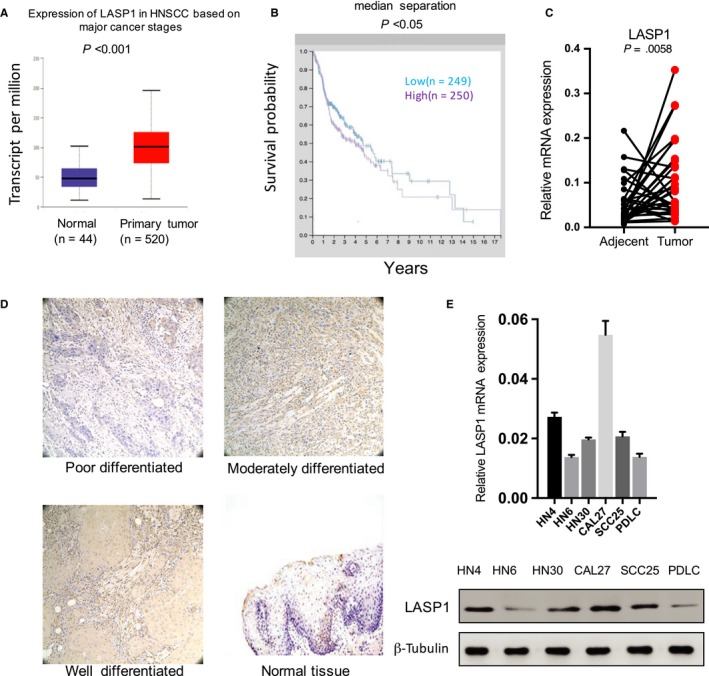
LASP1 is up‐regulated in HNSCC and associated with a poor prognosis. A, The difference in LASP1 expression between HNSCC tissues and normal adjacent tissues based on data from the UALCAN database (*P* < .001). B, The relationship between LASP1 expression and patient survival time based on data from the human protein Atlas database (*P* < .05). C, Real‐time PCR analysis of the mRNA expression of LASP1 in 40 pairs of HNSCC tissue specimens and the corresponding adjacent normal tissues (*P* = .0058). D, The representative IHC images of LASP1 expression in the well‐, moderately and poorly differentiated HNSCC tissues and adjacent normal tissues. E. Real‐time PCR and Western blot were used to detect the mRNA and protein expression levels of LASP1 in five HNSCC cell lines and PDLC cells (*P* < .001)

To examine the pattern of LASP1 expression in HNSCC tissues, we first retrieved the available public bioinformatics database. In the UALCAN database,^19^ the expression of LASP1 mRNA in HNSCC tissues was significantly higher than that in normal control tissues (Figure 1A). In the human protein Atlas database,^20^ the Kaplan‐Meier survival curve showed that the survival rate of patients with low expression of LASP1 was significantly higher than that of patients with high expression of LASP1 in highly differentiated HNSCC tissues, indicating poor prognosis in tumours with high expression of LASP1 (Figure 1B). We next examined the mRNA expression of LASP1 in 40 pairs of HNSCC tissue specimens that were collected in our medical institute. The levels of primary tumour LASP1 were clearly increased compared with those of the adjacent normal tissues, according to the real‐time PCR assay (Figure 1C). The mRNA expression of LASP1 in the HNSCC tissues was associated with lymph node metastasis (*P* = .0482) and TNM stage (*P* = .0174) but not age, sex or smoking history. The mRNA expression of HSPA1A was not associated with any clinical parameters (Table 1). The immunohistochemistry (IHC) results revealed that LASP1 was highly expressed in poorly differentiated HNSCC tissues compared with normal adjacent tissues, suggesting that the expression of LASP1 is positively correlated with the degree of differentiation of HNSCC (Figure 1D). We also observed that the mRNA and protein expression of LASP1 were high in the HNCSS cell lines compared with the PDLC cells, especially highest in CAL27 and lowest in HN6 (Figure 1E). These findings indicate that LASP1 is up‐regulated in HNSCC tissues and is associated with a poor prognosis, both CAL27 and HN6 were selected as represent cell lines for further study.

**Table 1:** Associations between LASP1, HSPA1A mRNA levels and clinical parameters in a training cohort (n = 40)

	LASP1 mRNA expression (2^−ΔΔCt^ Mean ± SD)	*P* value	HSPA1A mRNA expression (2^−ΔΔCt^ Mean ± SD)	*P* value
Age (y)				
≥60	0.3265 ± 0.1363	.2016	0.1729 ± 0.04684	.2667
<60	0.1234 ± 0.04475		0.11 ± 0.02279	
Gender				
Man	0.1494 ± 0.04484	.2027	0.1118 ± 0.02075	.7833
Women	0.4351 ± 0.2367		0.2211 ± 0.07715	
Smoking history				
Yes	0.1624 ± 0.0649	.4799	0.1494 ± 0.03399	.8963
No	0.2787 ± 0.1197		0.1417 ± 0.04018	
T stage				
T1‐T1	0.2461 ± 0.09555	.7836	0.1404 ± 0.02746	.7686
T3‐T4	0.1912 ± 0.09698		0.1613 ± 0.09072	
Lymph node metastasis				
pN positive	0.2781 ± 0.1277	.0482	0.1733 ± 0.04422	.2605
pN negative	0.1826 ± 0.08048		0.1095 ± 0.0296	
TNM stage				
I–II	0.1431 ± 0.07442	.0174	0.1138 ± 0.03107	.3487
III–IV	0.3031 ± 0.1245		0.1673 ± 0.04267	
Perineural invasion				
Yes	0.1324 ± 0.03466	.3984	0.1016 ± 0.0202	.3488
No	0.2791 ± 0.1107		0.1609 ± 0.03745	
ECS				
Yes	0.1108 ± 0.04467	.5560	0.111 ± 0.04269	.6544
No	0.2529 ± 0.08923		0.1494 ± 0.03134	
Prior radiotherapy				
Yes	0.1181 ± 0.06172	.5381	0.1241 ± 0.0168	.7621
No	0.2557 ± 0.09149		0.1482 ± 0.03271	

Note

The mRNA expression of LASP1 in head and neck tumour tissues was associated with lymph node metastasis (*P* = .0482) and TNM stage (*P* = .0174), but not with age, gender or smoking history. The mRNA expression of HSPA1A was not associated with any clinical parameters**.**

### 3.2 Knockdown of LASP1 in HNSCC cells inhibits cell proliferation, migration and invasion and enhances the cell cycle G2/M stage

**Figure 2:**
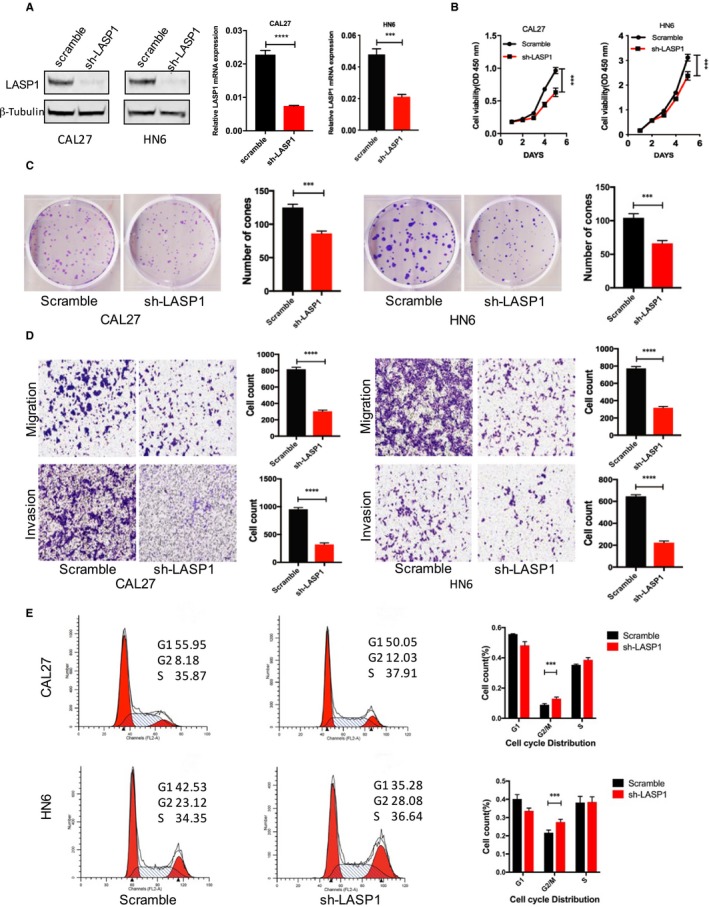
The knockdown of LASP1 in HNSCC cells inhibits cell proliferation, migration and invasion and enhances the G2/M stage of the cell cycle. A, Western blot and real‐time PCR were used to detect the LASP1 interference efficiency following the lentivirus‐shRNA infection of HNSCC cells. B, Cell proliferation at 6 d was measured by CCK‐8 following the LASP1 interference. C, The plate cloning formation assay was performed to determine colony‐forming ability after LASP1 interference; clones containing more than 50 cells were counted. D, A transwell analysis was performed to measure metastasis and invasion following LASP1 interference in the HNSCC cells. E, The representative FACS results indicate the cell cycle changes following the LASP1 interference. The quantitative results were analysed in the three independent experiments, *** *P* < .05, ****P < .01 compared with the control group

To investigate the effect of LASP1 interference on the biological function of HNSCC cells, CAL27 and HN6 cells were selected to be transduced with LASP1 shRNAs at the same time for rigorous study. The mRNA and protein levels of LASP1 were significantly decreased after the lentivirus infection (Figure 2A). In addition, the CCK‐8 proliferation data showed that interference with LASP1 significantly inhibited cell growth in both cell lines (Figure 2B). The plate cloning experiments revealed that the interference with LASP1 significantly inhibited the plate clone formation (Figure 2C). Moreover, LASP1 interference significantly inhibited metastasis and invasion in the transwell experiments (Figure 2D). We also determined that the G2/M phase of the CAL27 and HN6 cells increased significantly after LASP1 interference (*P* < .05), as detected by flow cytometry, whereas there was no significant change in the G1 and S phase, indicating that LASP1 promotes the cell cycle G2/M phase transition (Figure 2E).

### 3.3 Overexpression of LASP1 in HNSCC cells promotes cell proliferation, migration and invasion and enhances the cell cycle G2/M stage

**Figure 3:**
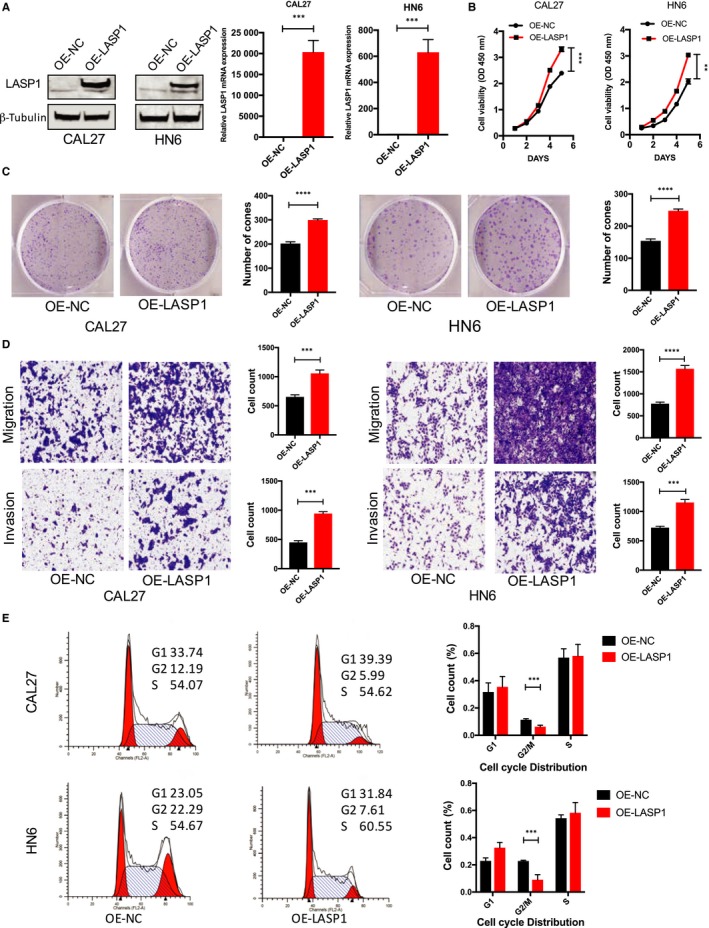
Overexpression of LASP1 in HNSCC cells promotes cell proliferation, migration and invasion and enhances the G2/M stage of the cell cycle. A, Western blot and real‐time PCR were used to detect the LASP1 overexpression efficiency after the lentivirus‐OERNA infection of HNSCC cells. B, Cell proliferation at 6 d was measured by CCK‐8 following the overexpression of LASP1. C, A plate cloning formation assay was performed to investigate colony forming after the overexpression of LASP1; the number of clones containing >50 cells was counted. D, A transwell analysis was performed to measure metastasis and invasion following the overexpression of LASP1 in the HNSCC cells. E, The representative FACS results indicate the cell cycle changes following the overexpression of LASP1. The quantitative results were analysed in three independent experiments, ***P < .05, ****P < .01 compared with the control group

To investigate the effects of LASP1 overexpression on the biological function of HNSCC cells, LASP1 was overexpressed in the CAL27 and HN6 cells at the same time, and it was determined that the exogenous LASP1 protein and mRNA levels were significantly increased after lentivirus infection (Figure 3A). In the CCK‐8 proliferation experiments, the overexpression of LASP1 significantly promoted cell growth (Figure 3B), promoted the plate cloning (Figure 3C) and enhanced metastasis and invasion (Figure 3D). In addition, the G2/M phase of the CAL27 and HN6 cells decreased significantly following the overexpression of LASP1 (*P* < .05), as measured with flow cytometry, whereas there was no significant change in the G1 and S phase. These findings indicate that LASP1 positively promotes the cell cycle G2/M phase transition (Figure 3E).

### 3.4 LASP1 promotes HNSCC migration and invasion in vivo

**Figure 4:**
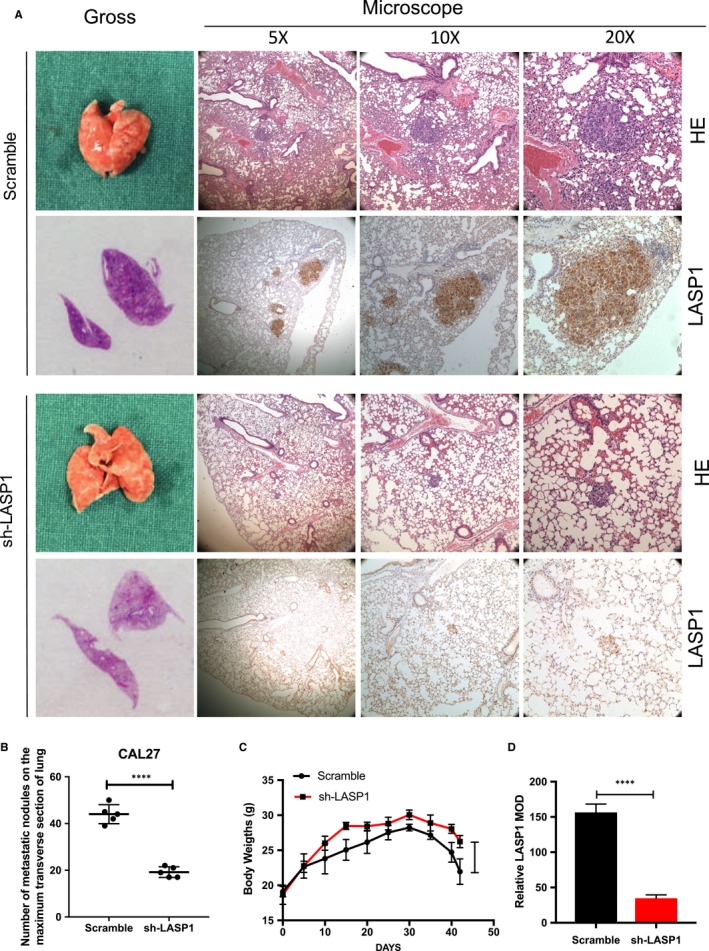
LASP1 overexpression promotes HNSCC cell migration and invasion in vivo. A, The representative images of lung tissues of nude mice. After the HNSCC CAL27 cells (1 × 10^6^) were injected into the nude mice via the tail vein for 42 d, the lung tissues were fixed and examined grossly and then under the microscope (5×, 10×, 20×). H‐E staining was performed, and IHC staining was used to detect the expression of LASP1 in metastatic lesions. B, The quantity of metastases node in the largest transverse section of the lungs in the nude mice (n = 5 per group). ****P < .01 compared with the Scramble group. C, The body weight change of the nude mice (n = 5 per group) ****P < .01 in the interference group compared with the Scramble group. D, The relative number of LASP1 MOD between the interference group and the Scramble group was observed using ImageJ software. (n = 5 per group) ****P < .01

To investigate the effect of LASP1 interference on HNSCC in vivo, CAL27 (sh‐LASP1) and control cells were injected into nude mice through the tail vein. After 42 days, we found that the number of surface nodules in the lung tissue in the interference group was significantly lower than that in the control group (Figure 4A). In addition, the body weight in the interference group was significantly higher than that in the control group (Figure 4C). H‐E staining of the lung cancer tissue metastasis tumour nest confirmed that LASP1 expression from tumour nodules in the interference group was lower than that of the control group. The mean value of metastatic nodules in the interference group was 46 ± 16.5, compared with 19.2 ± 5.2 in the control group (*P* < .001) (Figure 4B). The relative number of LASP1 MOD in the interference group was obvious lower compared with that of the Scramble group, as observed using ImageJ software (n = 5 per group) ****P < .01(Figure 4D).

### 3.5 LASP1 and HSPA1A directly and closely bind and co‐localize in the cytoplasm of HNSCC cells

**Figure 5:**
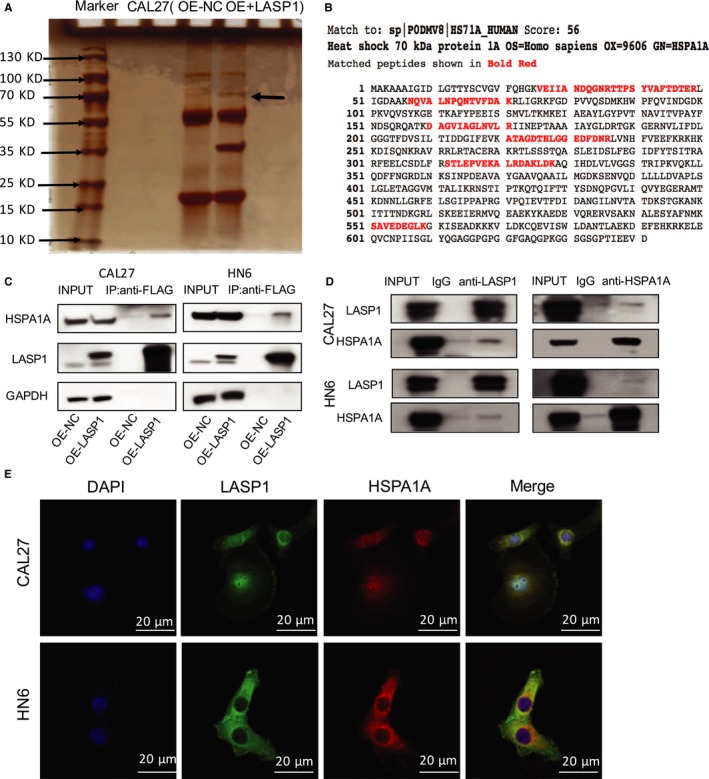
LASP1 and HSPA1A bind directly with each other and are co‐in the cytoplasm. A, A Co‐IP assay followed by LC/MS/MS examination was performed to detect the directly interactive binding partner of LASP1 in the CAL27 cells. The most obvious single band was detected at 70 kd. B, As indicated by mass spectrometry, the protein interactive with LASP1 was HSPA1A. C, The results of the co‐IP assay indicate that LASP1 directly binds with HSPA1A. D, The results of the co‐IP assay showed that HSPA1A is also closely interactive with LASP1. E, The subcellular localization of HSPA1A and LASP1 in the CAL27 cells was assessed by immunofluorescence staining

To identify the direct interactive partner of LASP1, we performed a Co‐IP assay followed by a LC/MS/MS examination. In the CAL27 cells, after we overexpressed FLAG‐tagged LASP1, we observed an obvious 70 KD silver‐stained band indicating the immunoprecipitated FLAG‐tag compared with the negative control lane (Figure 5A). This distinct band was identified as HSPA1A by mass spectrometry with a score of 56, and the matched peptides included VEIIANDQGNRTTPSYVAFTDTER, NQVALNPQNTVFDAK, DAGVIAGLNVLR, ATAGDTHLGGEDFDNR, STLEPVEKALRDAKLDK, SAVEDEGLK (Figure 5B). The interaction between LASP1 and HSPA1A was verified by Co‐IP assay in CAL27 and HN6 cells (Figure 5C). We then verified the endogenous protein interaction between HSPA1A and LASP1 in the cell lines. The cells were lysed and purified using an anti‐LASP1 or anti‐HSPA1A affinity gel. Then, the protein pellets were analysed by Western blot with anti‐LASP1 or anti‐HSPA1A (Figure 5D). We also used immunofluorescence staining to confirm the subcellular colocalization of HSPA1A and LASP1 in the cytoplasm (Figure 5E).

### 3.6 HSPA1A positively regulates the interaction of LASP1 with phosphorylated AKT

**Figure 6:**
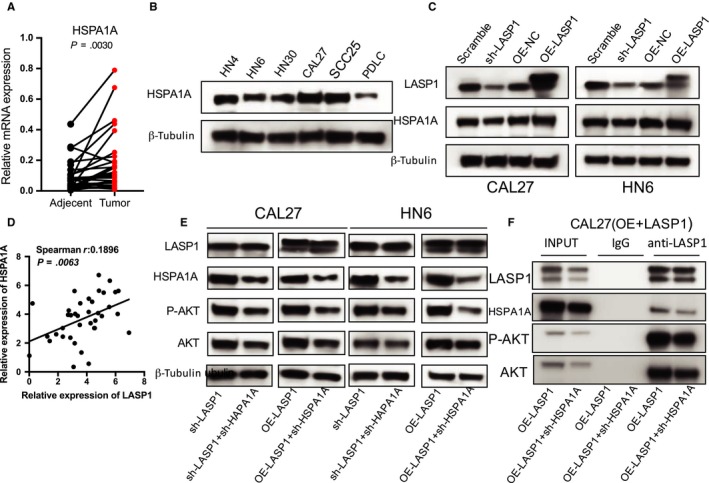
HSPA1A positively regulates LASP1‐pAKT directly binding. A, The real‐time PCR analysis of HSPA1A mRNA expression in 40 pairs of HNSCC tissue specimens and adjacent normal tissues (*P* = .0030). B, The Western blot analysis of HSPA1A levels in the HNSCC cell lines and PDLC cells. C, The Western blot analysis revealed that HSPA1A protein levels were significant altered in HNSCC cells (Scramble, sh‐LASP1, OE‐NC, OE‐LASP1). D, A significant positive correlation was observed between the LASP1 and HSPA1A expression levels in the HNSCC tissues (n = 40). E, Western blot analysis was performed to detect the HSPA1A interference efficiency and the influence of HSPA1A interference on the protein expression of P‐AKT and AKT. F, A Co‐IP analysis data were performed. The CAL27 (OE‐LASP1) cells were lysed and purified with an anti‐LASP1 affinity gel. The protein pellets were analysed by immunoblotting with anti‐LASP1, anti‐HSPA1A, anti‐P‐AKT and anti‐AKT. *** *P* < .05, **** *P* < .01

To examine the critical role of HSPA1A on LASP1 function in HNSCC, we first manipulated the levels of LASP1 and subsequently detected the HSPA1A protein expression level by Western blot assay. As indicated in Figure 6A, there was an obvious change in the levels of HSPA1A protein in the CAL27 and HN6 cells (Scramble, sh‐LASP1, OE‐NC, OE‐LASP1) (Figure 6B). In addition, the mRNA expression of HSPA1A in the 40 pairs of HNSCC specimens was obviously higher compared with the adjacent tissues, as measured with real‐time PCR, and there was a positive correlation between LASP1 and HSPA1A expression (Figure 6A, 6). The protein level of HSPA1A was also up‐regulated in the five HSNCC cell lines compared with the PDLC cells, according to the Western blot (Figure 6B), as well as the pattern of LASP1 (Figure 1E). Although the HSPA1A protein levels were significantly decreased by the lentivirus‐mediated interference, the protein levels of LASP1 were not obviously influenced. Meanwhile, the decrease in HSPA1A clearly reduced the P‐AKT levels (Figure 6E). To detect the interaction of LASP1 with AKT under the influence of HSPA1A, we also performed a Co‐IP assay with the CAL27 (OE‐LASP1) cellular lysis. We observed that the binding between LASP1 and P‐AKT was obviously decreased following HSPA1A interference (Figure 6F). These data indicate that the binding of HSPA1A did not affect the LASP1 stability but rather affected the interaction between LASP1 and P‐AKT.

**Figure 7:**
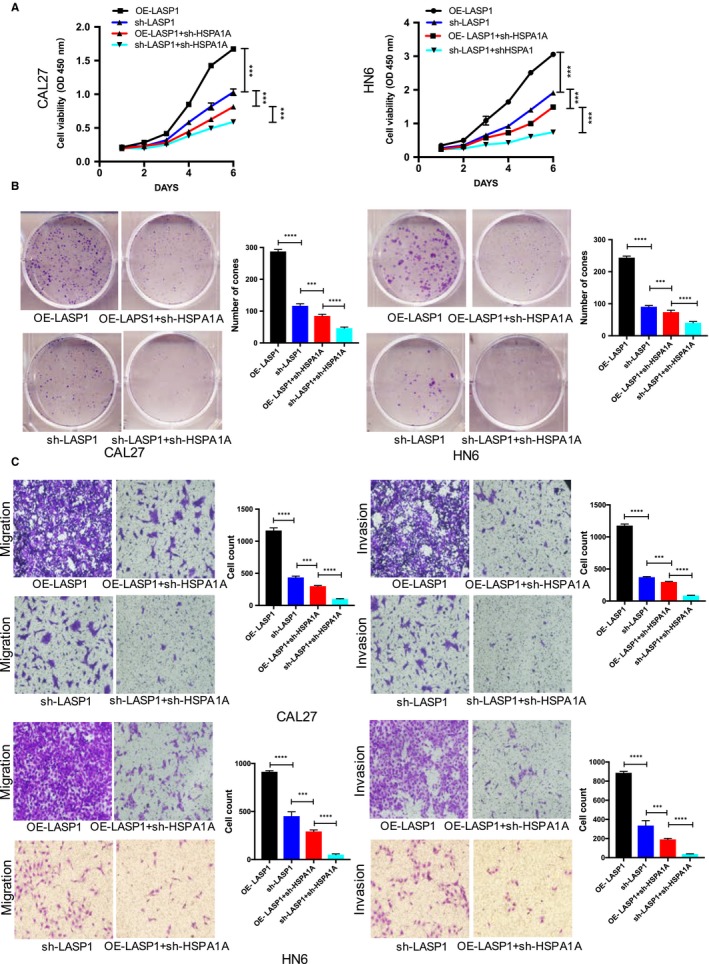
HSPA1A is essential for LASP1‐mediated HNSCC cell aggressiveness. A, Cell proliferation was measured by CCK‐8 in CAL27 cells at 6 days. B, The plate cloning formation assay was performed to determine the effect of HSPA1A interference on colony‐forming ability; clones with over 50 cells were counted. C, A transwell analysis was performed to investigate metastasis and invasion following the HSPA1A interference in the HNSCC cells. *** *P* < .05, **** *P* < .01

To investigate the effect of the coordination between LASP1 and HSPA1A on the biological behaviour of HNSCC cells, we performed a CCK‐8 proliferation assay. Higher levels of LASP1 in the CAL27 and HN6 (OE‐LASP1) cells significantly promoted cellular proliferation compared with lower cells (sh‐LASP1), whereas knocking down HSPA1A attenuated the influence of overexpressing LASP1 (Figure 7A). In agreement with the cellular proliferation results, the cloning formation result also revealed that the interference of HSPA1A significantly inhibited the LASP1‐mediated increase in clone formation (Figure 7B). Double‐knocking down LASP1 and HSPA1A was shown to be the most powerful treatment to inhibit cellular proliferation, metastasis and invasion (Figure 7C). These data indicate the critical role of HSPA1A on the biological function of LASP1 in HNSCC cells.

## 4 DISCUSSION

In this study, we examined LASP1 levels through a publicly available database and our native data. We observed that a high expression of LASP1 is closely related to shorter survival and a poor prognosis in HNSCC tissue. Furthermore, the overexpression of LASP1 promoted proliferation, the formation of cell clones, metastasis and invasion, the acceleration of the transition of G2/M phase in the HNSCC cell lines and the formation of lung metastasis in nude mice in vivo. To identify the binding partner of LASP1 that mediated the pro‐oncogenic effect, we performed a Co‐IP assay followed by LC/MS/MS examination. Interestingly, we confirmed that the heat shock protein family A member, HSPA1A, is the most significant binding partner of LASP1. Moreover, HSPA1A is critical for the promotion of the biological behaviour of HNSCC cell lines by enhancing the interaction between LASP1 and P‐AKT.

There is increasing evidence that LASP1 is not only a specific focal adhesion protein with related cytoskeletal proteins but also a signalling protein which performs its biological function with multiple chaperones, dynamically participating in a wide range of cellular behaviours.^21^, ^22^ While the well‐known protein AKT plays a crucial role in regulating survival via the phosphorylation of the internal carboxy terminus of Ser473,^23^, ^24^, ^25^ the importance of LASP1 on P‐AKT levels has not been well studied. Previous studies have shown that LASP1 mediates the PI3K/AKT pathway in many tumours. Depletion of LASP1 resulted in decreased P‐AKT levels, whereas the overexpression of LASP1 resulted in enhanced P‐AKT accumulation.^26^ In non‐small cell lung cancer, LASP1 promotes the progression to a malignant phenotype by inducing the phosphorylation of the FAK‐AKT pathway.^27^ LASP1 can promote the metastasis and invasion of nasopharyngeal carcinoma cells in vitro and negatively regulates the LASP1/PTEN/AKT axis.^28^ These studies support the notion that the LASP1/P‐AKT interaction is related to the biological behaviour of tumour cells.

Therefore, after we confirmed the direct binding of LASP1 with HSPA1A, we speculated the interaction between LASP1 and the P‐AKT/AKT pathway and that inhibiting HSPA1A would greatly reduce the biological behaviour of HNSCC cell lines. HSPA1A is widely present in the cytoplasm, exosomes and cell membrane, and this protein induces tumour cell growth, cell migration as well as therapeutic resistance.^29^ HSPA1A fulfils various functions in tumour cells and is overexpressed in many different tumour types.^30^ In colorectal cancer, specific pharmacological inhibitors or the gene knockdown HSPA1A is being used as a new treatment strategy.^31^ HSPA1A is also a potential prognostic factor for metastatic disease with advanced serous carcinoma.^32^ HSPA1A also plays a role in the PI3K/AKT pathway. For example, chronic MnSO_4_ exposure can activate the hippocampal PI3K/AKT signalling pathway in the rat hippocampus, leading to apoptosis of the central nervous cells, accompanied by enhanced HSPA1A transcription and translation.^33^ The PI3K inhibitor LY294002 effectively reduces the sensitivity of tumours by down‐regulating the expression of HSPA1A.^34^ We showed that doubly knocking down HSPA1A and LASP1 resulted in a much more obvious decrease in P‐AKT levels, suggesting a possible strategy to inhibit HNSCC progression.

In summary, LASP1 and HSPA1A are both up‐regulated in HNSCC, and HSPA1A directly binds to LASP1, which synergistically affects the development of HNSCC. We therefore expect that the double inhibition of LASP1 and HSPA1A expression may be an effective method for the treatment of HNSCC.

## CONFLICT OF INTEREST

The authors confirm that there are no conflicts of interests.

## ETHICS APPROVAL AND CONSENT TO PARTICIPATE

All animal experiments were approved by the Animal Research Committee of the Experimental Animal Research Center of the Ninth People's Hospital of Shanghai and in accordance with the guidelines for international animal research.

## CONSENT FOR PUBLICATION

This manuscript is consent for publication.

## Data Availability

The datasets used and/or analysed during the current study are available from the corresponding author on reasonable request.
